# Supplementary material for: Differences in transcription initiation directionality underlie distinctions between plants and animals in chromatin modification patterns at genes and cis-regulatory elements
Source: G3 (Bethesda). 2024 Jan 22;14(3):jkae016. doi: 10.1093/g3journal/jkae016 (PMC10917500; doi:10.1093/g3journal/jkae016)
Supplement: jkae016_Supplementary_Data [file jkae016_supplementary_data.pdf]

SUPPLEMENTARY FIGURES

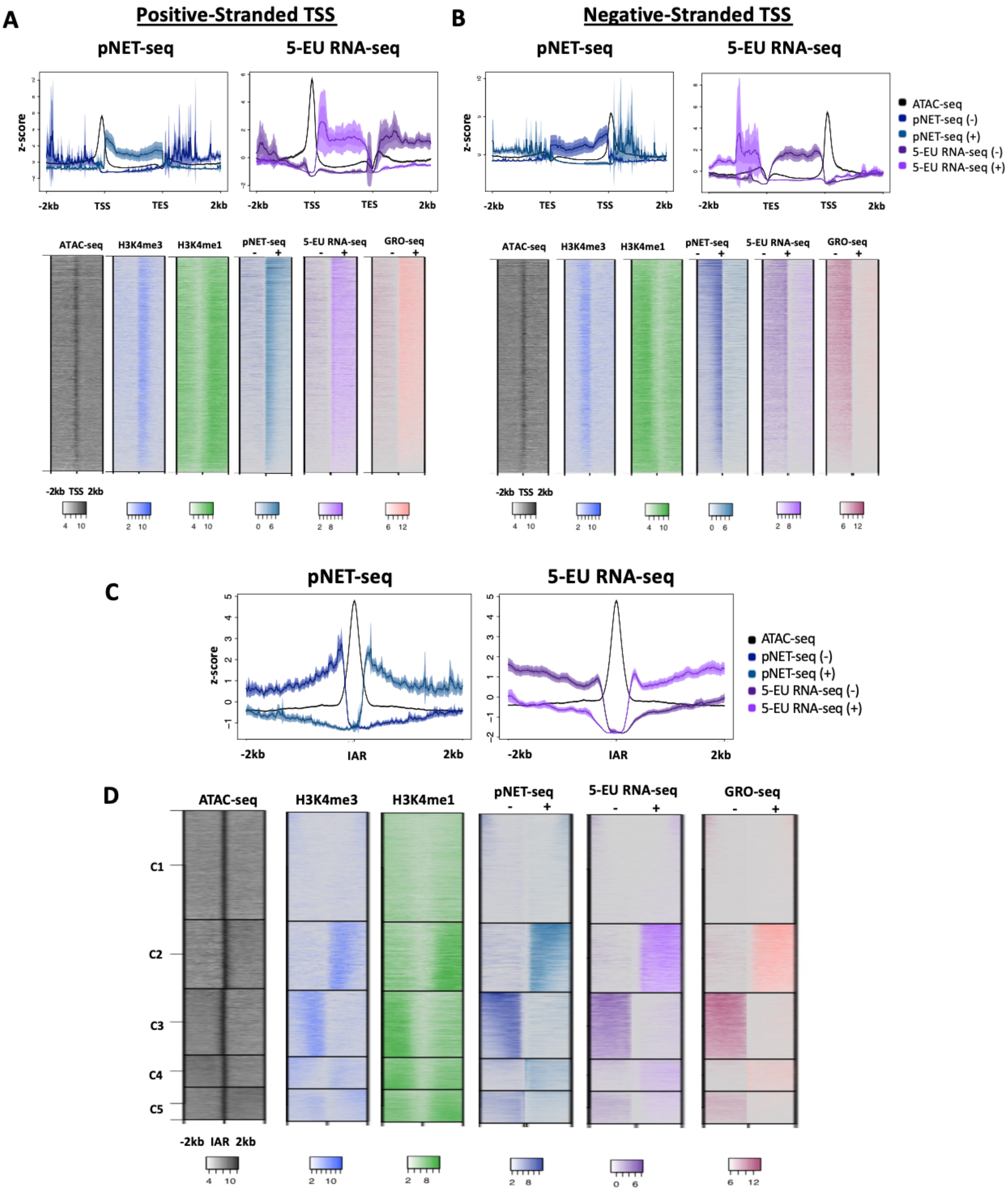

Supplementary Figure 1: Additional nascent RNA-seq datasets at TSSs and IARs in Arabidopsis thaliana. 5-EU RNA-seq and pNET-seq are shown as average plots over gene bodies (top images) at Arabidopsis positive-stranded TSSs (**A**) as well as negative-stranded TSSs (**B**). Solid lines represent the mean signal intensity (z-score transformed); the inner, dark-shaded region represents the standard error;

and the outer, light-shaded region represents the 95% confidence interval (CI) of signal intensity. Lower images in each panel show the nascent RNA data in heatmap form, along with ChIP-seq data for H3K4me1 and H3K4me3. (**C**). Average plots of nascent RNA signals at Arabidopsis intergenic accessible chromatin regions (accessible sites outside of gene bodies and 100-2000 bp upstream of a TSS). (**D**) Images in each panel are K-means clustered heatmaps containing all three types of nascent RNA data, along with ChIP-seq data for H3K4me1 and H3K4me3 at intergenic accessible regions.

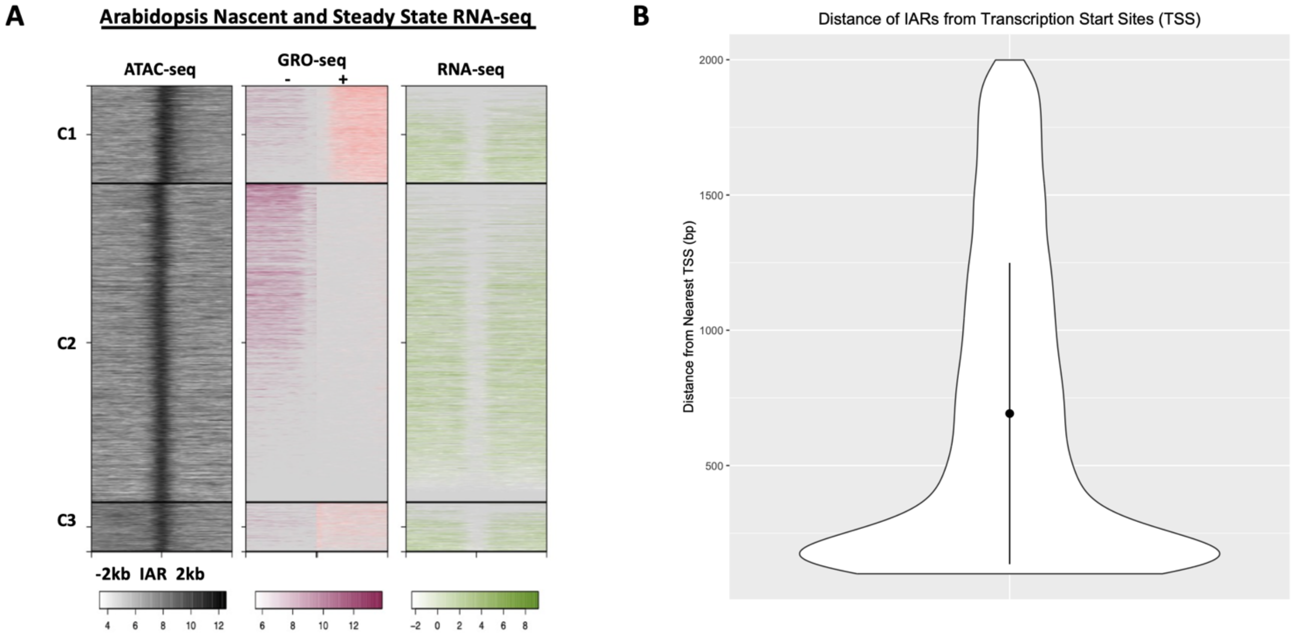

Supplementary Figure 2: Comparison of nascent and steady-state RNA-seq at intergenic enhancer regions in Arabidopsis. A) Clustered heatmaps of ATAC-seq, GRO-seq, and RNA-seq at intergenic accessible regions (IARs), which are defined as accessible chromatin sites outside of transcribed protein coding genes and within the range of 100-2000 bp from the nearest TSS. Windows extend 2 kb upstream and 2 kb downstream of the IARs; heatmaps have been log2 transformed. **B)** Distribution of distances between IARs and TSSs, showing an average distance of 692 bp from a TSS.

SUPPLEMENTARY TABLES

Supplementary Table 1: Publicly available dataset information

Species	Sample Type	Data Type	Data Source	Experiment Accession Number	Accession Number(s) of Raw Files Downloaded	File Type	Genome Version Used
Arabidopsis thaliana	Root epidermal non-hair cell nuclei	ATAC-seq	GEO	GSE101482	GSM2704265	.fastq	TAIR10
Arabidopsis thaliana	6-day old seedlings	GRO-seq	GEO	GSE83108	GSM2193124; GSM2193125	.fastq	TAIR10
Arabidopsis thaliana	12-day-old seedlings	pNET-seq	GEO	GSE109974	GSM2974949; GSM2974950	.fastq	TAIR10
Arabidopsis thaliana	5-day-old seedlings	5-EU RNA-seq	GEO	GSE118462	GSM3330492	.fastq	TAIR10
Arabidopsis thaliana	3rd and 4th rosette leaves	RNA-seq	*To be deposited (available on request)*				TAIR10
Homo sapiens	Common myeloid progenitor cell, CD34-positive female adult (27 yrs.)	ChIP-seq, H3K27Ac	ENCODE	ENCSR891KSP	ENCFF668OEU ; ENCFF641LUF ; ENCFF904CSC	.fastq	GRCh38.90
Homo sapiens	Common myeloid progenitor cell, CD34-positive female adult (27 yrs.)	ChIP-seq, H3K27Me3	ENCODE	ENCSR862NIZ	ENCFF279SSJ ; ENCFF399TRZ ; ENCFF410VSH ; ENCFF376VMQ ; ENCFF962LHH	.fastq	GRCh38.90
Homo sapiens	Common myeloid progenitor cell, CD34-positive female adult (27 yrs.)	ChIP-seq, H3K4Me1	ENCODE	ENCSR979YDQ	ENCFF186HNE ; ENCFF828SZM ; ENCFF886UDA ; ENCFF738ARX ; ENCFF376JZL	.fastq	GRCh38.90
Homo sapiens	Common myeloid progenitor cell, CD34-positive female adult (27 yrs.)	ChIP-seq, H3K4Me3	ENCODE	ENCSR850RTJ	ENCFF102IJI	.fastq	GRCh38.90
Homo sapiens	Common myeloid progenitor cell, CD34-positive female adult (27 yrs.)	ChIP-seq, Control for H3K4Me3	ENCODE	ENCSR707TMM	ENCFF599JOR ; ENCFF088FNX	.fastq	GRCh38.90
Homo sapiens	Common myeloid progenitor cell, CD34-positive female adult (27 yrs.)	ChIP-seq, Control for H3K4Me1, H3K27Ac, H3K27Me3	ENCODE	ENCSR919RJD	ENCFF606EYK ; ENCFF825IBW ; ENCFF054LZZ ; ENCFF168STH ;	.fastq	GRCh38.90
Homo sapiens	Common myeloid progenitor cell, CD34-positive female adult (27 yrs.)	DNase-seq	ENCODE	ENCSR122VUW	ENCFF164DKI ; ENCFF613FMP ; ENCFF776EIK ; ENCFF395CSF ; ENCFF175GQQ	.fastq	GRCh38.90
Homo sapiens	CD34+ erythrocytes	GRO-seq	GEO	GSE102819	GSM2746831 ; GSM2746829	.fastq	GRCh38.90
Drosophila melanogaster	S2 cells	DNase-seq	ENCODE	ENCSR834VXA	ENCFF005BHD	.fastq	Dm6
Drosophila melanogaster	S2 cells	ChIP-seq, H3K27Ac	GEO	GSE41440	GSM1017404 ; GSM1017405	.sra	Dm6
Drosophila melanogaster	S2 cells	ChIP-seq, H3K27Me3	GEO	GSE41440	GSM1017406	.sra	Dm6
Drosophila melanogaster	S2 cells	ChIP-seq, H3K4Me1	GEO	GSE41440	GSM1017407 ; GSM1017408	.sra	Dm6
Drosophila melanogaster	S2 cells	ChIP-seq, H3K4Me3	GEO	GSE41440	GSM1017409 ; GSM1017410	.sra	Dm6
Drosophila melanogaster	S2 cells	ChIP-seq, Control for H3K27Ac	GEO	GSE41440	GSM1017394 ; GSM1017395 ;	.sra	Dm6

Drosophila melanogaster	S2 cells	ChIP-seq, Control H3K27Me3	GEO	GSE41440	GSM1017397 ;	.sra	Dm6
Drosophila melanogaster	S2 cells	ChIP-seq, Control for H3K4Me1	GEO	GSE41440	GSM1017394 ; GSM1017397 ;	.sra	Dm6
Drosophila melanogaster	S2 cells	ChIP-seq, Control for H3K4Me3	GEO	GSE41440	GSM1017398 ; GSM1017399	.sra	Dm6
Drosophila melanogaster	S2 cells	GRO-seq	GEO	GSE23543	GSM577244	.fastq	Dm6
Oryza sativa	7-day-old leaf tissue	ATAC-seq; ChIP-seq	GEO	GSE128434	GSM3674604 ; GSM3674684 ; GSM3674685	.fastq	IRGSP-1.0
Glycine max	10-day-old leaf tissue	ATAC-seq; ChIP-seq	GEO	GSE128434	GSM3674586 ; GSM3674644 ; GSM3674645	.fastq	Glycine Max V1.0

Supplementary Table 2: *A. thaliana* ChIP-seq antibody information

Target	Antibody Name	Supplier	Concentration	Quantity Used per Reaction
H3K4me1	ab8895	Abcam	0.5 mg/mL	2 µg
H3K4me3	ab8580	Abcam	0.45 mg/mL	1.8 µg
H3K27ac	ab4729	Abcam	0.5 mg/mL	2 µg
H3K27me3	07-449	Millipore	0.5 mg/mL	2 µg
H3	ab1791	Abcam	0.5 mg/mL	2 µg

Supplementary Table 3: Data quality of *A. thaliana* root epidermal non-hair cell ChIP-seq datasets

Dataset type	Read size (nt)	Single end (SE) or paired end (PE)	Total reads	Total mapped reads	Total mapped q2 filtered reads	Total nuclear peaks called (via HOMER)	Avg. size of peaks (bp)	Std. dev. of peak size (+/- bp)	Median size of peaks (bp)
			(x 10^6)	(x 10^6)	(x 10^6)				
ChIP-seq (H3K4me1)	50	SE	83.2	73.1	54.7	31,016	402.54	201.26	330
ChIP-seq (H3K4me3)	50	SE	101.1	91.5	82.6	14,718	277.12	134.57	189
ChIP-seq (H3K27ac)	50	SE	131.6	115	92	36,146	299.09	161.41	235
ChIP-seq (H3K27me3)	50	SE	22.5	19.8	16.1	27,784	378.9	223.91	303
ChIP-seq (H2A.Z)	50	SE	123.3	104.6	81.8	30,594	255.62	115.61	183
ChIP-seq (H3)	50	SE	62	53.8	34.3	23,379	254.38	76.43	211

Arabidopsis: Downstream				Arabidopsis: Upstream			
Cluster ID	Mean	Median	Standard Dev	Cluster ID	Mean	Median	Standard Dev
1	129	5.44	328	1	1661	1259	1272
2	65.6	5.44	267	2	84.4	8.74	311
3	416	151	678	3	74.9	8.74	241
4	1257	859	1133	4	145	8.74	387
5	63.2	5.44	208	5	543	204	785
Drosophila: Downstream				Drosophila: Upstream			
Cluster ID	Mean	Median	Standard Dev	Cluster ID	Mean	Median	Standard Dev
1	173	19.6	572	1	2143	1451	1983
2	131	0	665	2	59.5	0	397
3	1721	1126	1769	3	120	19.1	359
4	173	30	404	4	1277	703	1467
5	1870	1467	1348	5	1844	1475	1441
Human: Downstream				Human: Upstream			
Cluster ID	Mean	Median	Standard Dev	Cluster ID	Mean	Median	Standard Dev
1	40.1	36.6	32.5	1	9.7	0	20
2	4.44	0	12.4	2	8.24	0	18.4
3	13.4	4.65	22	3	139	119	71.1
4	69.3	61.5	35.1	4	12.1	5.62	19.2
5	183	167	65.5	5	19.9	11.2	28.6

Supplementary Table 4. Summary statistics for raw GRO-seq signal enrichment up and downstream of IARs for each species. Resulting un-transformed values of GRO-seq signal enrichment for each cluster, corresponding to Figure 3.
